# The links between chromatin spatial organization and biological function

**DOI:** 10.1042/BST20130213

**Published:** 2013-11-20

**Authors:** Alejandro Rodriguez, Pernilla Bjerling

**Affiliations:** *Department of Medical Biochemistry and Microbiology (IMBIM), Science for Life Laboratory, Uppsala University, Box 582, SE-751 23 Uppsala, Sweden

**Keywords:** chromatin, fission yeast, heterochromatin, nuclear organization, transcriptional regulation, 3C, chromosome conformation capture, 4C, circularized chromosome conformation capture, 5C, carbon copy chromosome conformation capture, CENP, centromere protein, ChIP, chromatin immunoprecipitation, CT, chromosome territory, Dam, DNA adenine methyltansferase, DamID, DNA adenine methyltransferase identification, FISH, fluorescence *in situ* hybridization, HiC, genome-wide chromosome conformation capture, INM, inner nuclear membrane, LAD, lamina-associated domain, LEM, Lap2/emerin/Man1, MPS, massive parallel sequencing, NAD, nucleoli-associated domain, NM, nuclear membrane, ONM, outer nuclear membrane, TFIIIC, transcription factor IIIC, ToR, time of replication

## Abstract

During the last few years, there has been a rapid increase in our knowledge of how chromatin is organized inside the nucleus. Techniques such as FISH (fluorescence *in situ* hybridization) have proved that chromosomes organize themselves in so-called CTs (chromosome territories). In addition, newly developed 3C (chromatin conformation capture) techniques have revealed that certain chromosomal regions tend to interact with adjacent regions on either the same chromosome or adjacent chromosomes, and also that regions in close proximity are replicated simultaneously. Furthermore, transcriptionally repressed or active areas occupy different nuclear compartments. Another new technique, named DamID (DNA adenine methyltransferase identification), has strengthened the notion that transcriptionally repressed genes are often found in close association with the nuclear membrane, whereas transcriptionally active regions are found in the more central regions of the nucleus. However, in response to various stimuli, transcriptionally repressed regions are known to relocalize from the nuclear lamina to the interior of the nucleus, leading to a concomitant up-regulation of otherwise silenced genes. Taken together, these insights are of great interest for the relationship between chromosomal spatial organization and genome function. In the present article, we review recent advances in this field with a focus on mammalian cells and the eukaryotic model organism *Schizosaccharomyces pombe*.

## Methods for studying nuclear organization

Before the advent of high-throughput molecular biology methods, microscopy was the main method to study the arrangement of chromosomes within the cell nucleus. Molecular biology methods such as ChIP (chromatin immunoprecipitation) are the main approach of studying the interaction between proteins and specific genomic sites. Although microscopy techniques and ChIP are still widely used, the newly developed technique of 3C (chromosome conformation capture) has dramatically increased the observational resolution with respect to genome properties and the nuclear organization of chromosomes [1].

### Microscopy

Several different microscopy approaches, including light, electron and fluorescence microscopy, are available today. In this section, we focus on fluorescence-based techniques with respect to the study of nuclear architecture. The nuclear organization is determined through the establishment of reference point positions within the nucleus such as the NM (nuclear membrane), the nucleolus or a certain chromatin region. In studies assessing movement of chromatin from one nuclear compartment to another, nuclear structures might not only be relevant as a reference point, but also indicate a biological function [2,3].

For live-cell imaging purposes, generating a fusion between the protein of interest and a fluorescent tag is an important approach to study chromatin organization. GFP is the most widely used fluorochrome, but other alternatives with different emission wavelengths have emerged in recent years. The resulting hybrid proteins can be visualized directly under the microscope at the same time as stimuli and/or growth conditions are altered, allowing for real-time analysis of relocalization events within the nucleus. Live-cell imaging can also be used to follow the position of a specific locus in the genome. This is done by the integration of *lacO* repeats into a specific genomic locus together with the integration at another site with a LacR–GFP fusion protein that is able to bind to the *lacO* repeats, thereby creating a detectable GFP signal from the locus of interest [4,5].

FISH (fluorescence *in situ* hybridization) is based on fluorescently labelled oligonucleotide probes that bind complementarily to either DNA or RNA. This method cannot be used for live-cell imaging purposes, since cells must be fixed before hybridization with the probe. It has nevertheless revolutionized our understanding of nuclear architecture. With refinements of the technique such as usage of multiple fluorescently labelled probes in combination with advanced image analysis, 3D structures can be generated. In one study, all 24 human chromosomes could be labelled in the nucleus using a technique of combinatorial labelling on the basis of the usage of seven fluorochrome sets [6].

### ChIP

ChIP is the most commonly used technique to study the association between well-defined proteins and genomic regions. The basic approach is the cross-linking of proteins with DNA, for example using formaldehyde, followed by shearing of the chromatin into soluble particles. Subsequently, the protein–DNA complex is pulled down using an antibody with affinity to the protein of interest. When the DNA-binding sequences are known and a comparison is sought between different conditions, the pulled-down DNA can be analysed by PCR. For whole-genome analysis purposes, however, the ChIP technique is now combined with high-throughput techniques such as microarrays (ChIP-chip) or sequencing (ChIP-seq) by newly developed MPS (massive parallel sequencing) techniques. This high-throughput approach has allowed for a wide coverage of protein–DNA interactive components of the human epigenome (http://www.roadmapepigenomics.org).

### Chromosome conformation capture

The 3C methods are primarily focused on structure and interactions at the intra- and inter-organizational level of chromosomes and chromosome regions. 3C was first reported in 2002, and variants of the method have been developed since such as 4C (circularized chromosome conformation capture), 5C (carbon copy chromosome conformation capture) and HiC (genome-wide chromosome conformation capture) [1]. These methods aim to chart long-range chromatin interactions, and the main goal is to generate a 3D representation of chromosomes on the basis of an estimation of the frequency at which different genomic loci are in close proximity to each other. The initial steps include fixation that cross-links the chromatin, followed by, for most purposes, restriction enzyme digestion. The fragmentation, in turn, generates shorter complexes of protein and DNA, and the DNA is then ligated in order to combine sequences that are in close spatial proximity to each other. In a following step, a 3C library is generated with short DNA sequences containing the restriction site in the middle. In the initial 3C approach, the generation of a matrix of interaction frequencies was carried out with either semi-quantitative or quantitative PCR using a range of primer pairs. The aim of this approach is the interactive relationship between two genomic loci. In the 4C approach, one locus of interest is screened against multiple other positions over the whole genome. Analysis is performed with either microarray technology or MPS. In the 5C approach, multiple genomic loci are screened against each other in order to generate shorter-interaction, usually below 1 Mb, frequency matrices. From these data, chromosome conformation can be predicted and visualized in a 3D fashion. The HiC methodology is similar to that of 5C, but the comparison is genome-wide [7].

### DamID (DNA adenine methyltransferase identification)

The DamID method is used to determine association of chromatin to any protein, making use of the enzyme Dam (DNA adenine methyltansferase) from *Escherichia coli* that creates a DNA modification, namely DNA adenine methylation, normally not present in eukaryotic cells [8]. Briefly, a fusion protein is generated between Dam and a protein of interest, resulting in the methylation of all the genomic loci positioned in the vicinity of the fusion protein. In the next step, the methylated regions are amplified using PCR and analysed by high-throughput techniques, i.e. microarrays or sequencing.

## Chromatin

The DNA in the nucleus is highly compacted and organized, in order to enable it to fit within the boundary of the cell nucleus. A necessity considering that the DNA molecules from a single cell, should they be stretched out, would together reach a length of 2 m. The basic units of organization are the nucleosomes, which are spaced in an even array throughout the genome, like beads on a string.

### Nucleosomes

The nucleosome consists of a stretch of 146 bp of DNA wrapped 1.65 turns around a histone octamer [9]. In addition to the canonical histones H2A, H2B, H3 and H4 building up the octamer, there is a wide range of histone variants present in nucleosomes at specific genomic loci whose presence affects the biological function in various ways. The majority of the histone variants reported have been found to replace H3 and H2A, for example CENP-A (centromere protein A). This well-studied histone variant is conserved from yeast to humans and substitutes for histone H3. It is involved in the kinetochore assembly at the central core centromere and thus plays an important role in chromosome segregation during mitosis and meiosis [10]. Another histone variant, H2A.Z, reported to be involved in many biological processes, replaces the canonical histone H2A at certain sites [11]. Among the many functions of H2A.Z, it has been suggested that the exchange of this histone from nucleosomes facilitates DNA double-strand break repair, but it has also been proposed to be involved in the differentiation of embryonic stem cells through interactions with PcG (polycomb group) proteins. In *Schizosaccharomyces pombe*, this histone variant has been implicated to play a role in chromosome segregation and centromeric silencing [12].

The N-terminal tails of the histones protrude out from the histone octamer and are subjected to post-translational modifications that dictate the accessibility to the DNA by, e.g., transcription factors. Some post-translational modifications can also be inherited through multiple cell divisions in a manner that allows distinct chromosomal loci to retain their specific signature of epigenetic marks [13]. The most commonly targeted amino acids are lysine and arginine, and the most studied modifications are methylation, acetylation, ubiquitylation, SUMOylation and phosphorylation, but many others have discovered, such as glycosylation and crotonylation [14,15].

### Euchromatin and heterochromatin

Euchromatin is characterized by high gene density and is commonly more accessible to transcriptional activation compared with heterochromatin. Histones within euchromatic areas are characterized by acetylation and by H3K4me2/3 (di/tri-methylated histone H3 Lys^4^). Heterochromatin, on the other hand, is characterized by low transcriptional activity, low abundance of genes and enrichment in repetitive DNA sequences. Furthermore, at the chromatin level, heterochromatin is hypoacetylated and has high amounts of H3K9me2/3 (di/tri-methylated histone H3 Lys^9^). This histone modification is in turn important for the recruitment of HP1 (heterochromatin protein 1), called Swi6 in *S. pombe*, and binds to H3K9me2/3 with its chromodomain. The binding to chromatin induces a conformational switch in Swi6 that drives oligomerization and subsequent spreading of heterochromatin [16]. In addition, in fission yeast, it is clear that RNAi is involved in heterochromatin formation, a process that is dependent on the proteins Ago1, Dcr1 and Rdp1 [17].

Heterochromatin can be divided further into constitutive and facultative heterochromatin. The latter is highly compacted and is defined by a more or less constant epigenetic signature and is found at chromosomal structures such as telomeres and centromeres where it assembles on repetitive DNA sequences. Facultative heterochromatin, on the other hand, is more abundant in terms of gene content and changes in chromatin state, i.e. transcriptionally active compared with state, can occur.

### Higher-order chromatin organization

**Figure 1:**
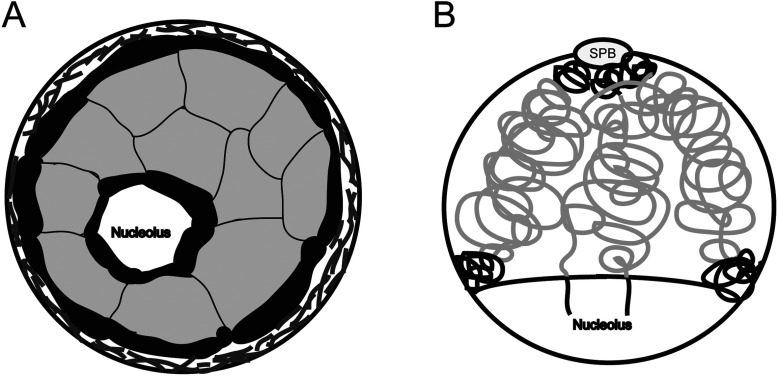
Schematic representation of the organization of (A) the human or (B) the fission yeast cell nucleus Transcriptionally active chromatin (grey) is localized in the central part of the nucleus and transcriptionally repressed (black) towards the nuclear periphery and in the vicinity of the nucleolus. (**A**) The nuclear membrane is coated by lamina proteins (dark grey) and the chromosomes are organized into distinct CTs. (**B**) The spindle pole body (SPB) is inserted into the nuclear membrane, and repressed heterochromatin is found next to the SPB and other transcriptionally repressed regions cluster in foci at the opposite end of the nucleus where the nucleolus is found.

The linear arrangement of nucleosomes comprises the most basic order of chromatin called the 11 nm fibre. This is then probably followed by the formation of a 30 nm fibre that will subsequently organize itself within the limits of each chromosome in the cell nucleus. Very little is known about the driving forces behind chromatin organization, but molecular crowding and interaction with the nuclear periphery seem to be contributing factors [8,18]. The chromatin is surrounded by the NM that is composed of an INM (inner nuclear membrane) and an ONM (outer nuclear membrane). In higher eukaryotes, the INM has a nuclear lamina composed of intermediate filaments, lamins and associated proteins, in contact with chromatin [8] (Figure 1A). Lamins are lacking in fission yeast, but there are the INM proteins Lem2 and Man1, which contain the conserved LEM (Lap2/emerin/Man1) domain [19]. Moreover, these two proteins have overlapping functions together with another IMN protein named Ima1, and all three proteins contacts chromatin [19–21].

In addition, genes encoding tRNA are also involved in organizing the chromatin, since, in both mammalian cells and *S. pombe*, tRNA genes have been shown to co-localize [22,23]. Moreover, there is growing evidence for TFIIIC (transcription factor IIIC), which is necessary for the transcription of tRNA by RNA polymerase III, to be involved in chromatin organization in mammalian as well as in yeast cells. There is a substantial portion of TFIIIC-bound loci devoid of polymerase III both in human and *S. pombe* cells [24,25]. In *S. pombe*, these TFIIIC-bound loci cluster at the nuclear periphery [24].

Finally, repetitive DNA sequences in the genomes contribute to the organization within the cell nucleus. Heterochromatin is found at repetitive sequences, and these tend to cluster together [26]. In *S. pombe*, the 13 full-length Tf2 retrotransposons, found at different positions along the DNA co-localize at one to three nuclear spots in the cell nucleus via a special type of chromatin formed by conserved CENP-B proteins and the methyltransferase Set1 [27].

## Functional genome organization

The nuclear organization of eukaryotic organisms is highly complex and dynamic. Besides the various structural components in the nucleus, the intra- and inter-chromosomal interactions, as well as contacts between chromatin and nuclear components is not static, but is steadily changing in response to environmental cues such as shifting availability of nutrients and developmental processes [2,3,28].

### Transcriptional activation

The chromosomal arrangement in the nucleus is organized into an intricate 3D structure. The interactions between different chromosomal regions play an important role in gene regulation. Mammalian chromosomes occupy discrete areas in the cell nucleus, called CTs (chromosome territories) (Figure 1A). They can be visualized by FISH techniques where unique probes for each chromosome are designed to individually paint the chromosomes [6]. In fission yeast, visualizing chromosome I and II also revealed distinct localization of the chromosomes [29] (Figure 1B). In addition, mapping the DNA interactions by 3C technologies strengthened further the existence of CTs in fission yeast [30]. Despite the defined organization of chromosomes into CTs, looping out of genomic loci does occur. This can happen when transcriptionally active loci translocate to spots in the cell nucleus with high concentrations of polymerase II, so-called transcription factories, at least in erythroid progenitor cells [31]. In *S. pombe*, there is evidence that genes regulated by the same transcription factor tend to co-localize, indicating the existence of transcription factories in this yeast [30].

### Correlation between replication timing and chromosome organization

Investigating the 3D organization of the DNA in the mammalian cell nucleus by 3C technologies has demonstrated that the chromatin folds into discrete globules of a few hundreds of kilobases [32,33]. Moreover, these domains have a distinct ToR (time of replication) [34,35]. These findings fit very well with the long-term notion that mammalian DNA replication occurs in replication factories that changes positions throughout the S-phase in a distinct spatiotemporal pattern [36]. Although the functional consequences of this organizational aspects is not clear at this point, there is some experimental evidence that some domains change ToR throughout development, which is accompanied by a change in subnuclear localization [37] (see below). Interestingly, the distinct spatiotemporal pattern of DNA replication is also evident in fission yeast [38].

### Gene silencing at the NM

The positioning of the chromosomes in relation to the NM and other structures is of great importance when it comes to gene expression. With the help of DamID, the LADs (lamina-associated domains) in the human genome were found to be enriched in transcriptionally repressed genes [8] (Figure 1A). Furthermore, these regions tend to be flanked by gene promoter regions that point away from the LADs, CpG islands and the protein CTCF (CCCTC-binding factor). When DamID was used to study LADs in single cells, it was observed that the contact of LADs to the nuclear lamina is dependent on the activity of the methyl H3K9 methyltransferase G9a and levels of H3K9me2 [39]. Furthermore, in mammalian cells, the part of the genome associated with the nucleolus, NADs (nucleoli-associated domains) are enriched in poorly expressed genes. Not surprisingly, an overlap between the LADs and the NADs has been reported indicating a similar subnuclear environment of transcriptional repression at the nuclear periphery as well as in the vicinity of the nucleolus [40]. Interestingly, there was a clear correlation between LADs and late ToR. In addition, if the replication time changed during development, there was also a change in the localization with respect to the nuclear lamina [37]. Moreover, in a recent study of chromatin using a large collection of different human cell types, several distinct chromatin profiles were identified: one transcriptionally active and several transcriptionally inactive clusters, one of them high in H3K9me3 [28]. Furthermore, the inactive chromatin states were enriched for contacts with the nuclear lamina [8]. Interestingly, the study by Zhu et al. [28] indicated that *in vitro* culturing in itself could promote the formation of heterochromatic regions with H3K9me3, and thus NM association, most probably due to a response to serum.

In *S. pombe*, transcriptionally repressed regions enriched in H3K9me2/3, such as centromeres, telomeres and mating type region, are localized to the nuclear periphery [41] (Figure 1B). In the case of centromeres, they are attached to the NM via Sad1, a SUN (Sad1 and UNC-84) domain protein inserted into the INM that in turn connects to Ksm1, containing a conserved KASH (Klarsicht, ANC-1 and Syne/Nesprin homology) domain, in the ONM. The Sad1–Ksm1 partners have been shown to have overlapping functions with a protein without any conserved domains called Csi1 in *S. pombe* [42,43]. Telomeres are attached to the NM by the connection of several proteins. Bqt3 is inserted into the NM and contacts Bqt4, which in turn binds Rap1 that finally binds Taz1 bound to the telomeric repeat region [44]. Moreover, both LEM domain-containing proteins, i.e. Man1 and Lem2, are necessary for the proper attachment of telomeres to the nuclear periphery, and Man1 was shown using DamID to be in contact with the subtelomeric regions [19,21]. Interestingly, the attachment of telomeres to the NM is not necessary for telomere integrity or function, since in a *bqt4*Δ mutant background, telomeres are no longer bound to the NM without any obvious consequences for telomere length or silencing. However, the detachment of telomeres during mitosis by the phosphorylation of Rap1 by Cdc2 and other kinases is critical for proper chromosome segregation [45].

Finally, in a recent study of Atf1-bound stress-response genes, these loci were shown to associate with the nuclear pores together with Dcr1 and other components of the RNAi pathway in *S. pombe*, thereby allowing the degradation of transcripts under non-induced conditions [46]. Another study also revealed the localization of gene clusters induced by nitrogen starvation to be localized to the NM under non-expressed conditions and to translocate to a more interior nuclear position upon activation [2]. Moreover, during induction and translocation, a drastic fall in nucleosome density over the gene bodies was detected [47].

## Future perspectives

The correlation between subnuclear localization and transcription has been established in a wide range of organisms. In response to environmental cues, there is a physical relocalization of chromosomal regions between the nuclear periphery and nuclear interior. What remains to be elucidated is whether the movement is a cause or a consequence of transcriptional activation. This question could be addressed by utilizing live-cell imaging combined by tethering chromosomal loci to the NM, preventing the movement, and at the same time quantifying gene expression in the absence or presence of an environmental cue. Another unresolved issue is what drives the movement of chromosomes; is the movement stochastic or motor-driven?

## Concluding remarks

The nuclear organization of eukaryotes exhibits an ordered structure and evolutionary conservation. In the present article, we have touched upon different aspects of how the nuclear organization of chromatin is associated with transcription and replication, with a focus on studies conducted in fission yeast and humans. Studies in these two evolutionarily distant organisms show that the distribution of chromatin and chromosomes are non-random. There is a distinction in spatial proximity of different chromosomal regions with respect to the NM. This distinction is reflected in differences of transcription levels and control of replication timing.

## References

[1] Dekker J., Marti-Renom M.A., Mirny L.A. (2013). Exploring the three-dimensional organization of genomes: interpreting chromatin interaction data. Nat. Rev. Genet..

[2] Alfredsson-Timmins J., Kristell C., Henningson F., Lyckman S., Bjerling P. (2009). Reorganization of chromatin is an early response to nitrogen starvation in *Schizosaccharomyces pombe*. Chromosoma.

[3] Peric-Hupkes D., Meuleman W., Pagie L., Bruggeman S.W., Solovei I., Brugman W., Graf S., Flicek P., Kerkhoven R.M., van Lohuizen M. (2010). Molecular maps of the reorganization of genome–nuclear lamina interactions during differentiation. Mol. Cell.

[4] Bjerling P., Olsson I., Meng X. (2012). Quantitative live cell fluorescence-microscopy analysis of fission yeast. J. Visualized Exp..

[5] Robinett C.C., Straight A., Li G., Willhelm C., Sudlow G., Murray A., Belmont A.S. (1996). *In vivo* localization of DNA sequences and visualization of large-scale chromatin organization using lac operator/repressor recognition. J. Cell Biol..

[6] Bolzer A., Kreth G., Solovei I., Koehler D., Saracoglu K., Fauth C., Muller S., Eils R., Cremer C., Speicher M.R. (2005). Three-dimensional maps of all chromosomes in human male fibroblast nuclei and prometaphase rosettes. PLoS Biol..

[7] de Wit E., de Laat W. (2012). A decade of 3C technologies: insights into nuclear organization. Genes Dev..

[8] Guelen L., Pagie L., Brasset E., Meuleman W., Faza M.B., Talhout W., Eussen B.H., de Klein A., Wessels L., de Laat W. (2008). Domain organization of human chromosomes revealed by mapping of nuclear lamina interactions. Nature.

[9] Luger K., Mader A.W., Richmond R.K., Sargent D.F., Richmond T.J. (1997). Crystal structure of the nucleosome core particle at 2.8 Å resolution. Nature.

[10] Allshire R.C., Karpen G.H. (2008). Epigenetic regulation of centromeric chromatin: old dogs, new tricks?. Nat. Rev. Genet..

[11] Skene P.J., Henikoff S. (2013). Histone variants in pluripotency and disease. Development.

[12] Hou H., Wang Y., Kallgren S.P., Thompson J., Yates J.R., Jia S. (2010). Histone variant H2A.Z regulates centromere silencing and chromosome segregation in fission yeast. J. Biol. Chem..

[13] Xu M., Wang W., Chen S., Zhu B. (2012). A model for mitotic inheritance of histone lysine methylation. EMBO Rep..

[14] Sakabe K., Wang Z., Hart G.W. (2010). β-*N*-acetylglucosamine (O-GlcNAc) is part of the histone code. Proc. Natl. Acad. Sci. U.S.A..

[15] Tan M., Luo H., Lee S., Jin F., Yang J.S., Montellier E., Buchou T., Cheng Z., Rousseaux S., Rajagopal N. (2011). Identification of 67 histone marks and histone lysine crotonylation as a new type of histone modification. Cell.

[16] Canzio D., Liao M., Naber N., Pate E., Larson A., Wu S., Marina D.B., Garcia J.F., Madhani H.D., Cooke R. (2013). A conformational switch in HP1 releases auto-inhibition to drive heterochromatin assembly. Nature.

[17] Volpe T.A., Kidner C., Hall I.M., Teng G., Grewal S.I., Martienssen R.A. (2002). Regulation of heterochromatic silencing and histone H3 lysine-9 methylation by RNAi. Science.

[18] Richter K., Nessling M., Lichter P. (2008). Macromolecular crowding and its potential impact on nuclear function. Biochim. Biophys. Acta.

[19] Gonzalez Y., Saito A., Sazer S. (2012). Fission yeast Lem2 and Man1 perform fundamental functions of the animal cell nuclear lamina. Nucleus.

[20] Hiraoka Y., Maekawa H., Asakawa H., Chikashige Y., Kojidani T., Osakada H., Matsuda A., Haraguchi T. (2011). Inner nuclear membrane protein Ima1 is dispensable for intranuclear positioning of centromeres. Genes Cells.

[21] Steglich B., Filion G.J., van Steensel B., Ekwall K. (2012). The inner nuclear membrane proteins Man1 and Ima1 link to two different types of chromatin at the nuclear periphery in *S. pombe*. Nucleus.

[22] Raab J.R., Chiu J., Zhu J., Katzman S., Kurukuti S., Wade P.A., Haussler D., Kamakaka R.T. (2012). Human tRNA genes function as chromatin insulators. EMBO J..

[23] Iwasaki O., Tanaka A., Tanizawa H., Grewal S.I., Noma K. (2010). Centromeric localization of dispersed ol III genes in fission yeast. Mol. Biol. Cell.

[24] Noma K., Cam H.P., Maraia R.J., Grewal S.I. (2006). A role for TFIIIC transcription factor complex in genome organization. Cell.

[25] Moqtaderi Z., Wang J., Raha D., White R.J., Snyder M., Weng Z., Struhl K. (2010). Genomic binding profiles of functionally distinct RNA polymerase III transcription complexes in human cells. Nat. Struct. Mol. Biol..

[26] Papait R., Pistore C., Grazini U., Babbio F., Cogliati S., Pecoraro D., Brino L., Morand A.L., Dechampesme A.M., Spada F. (2008). The PHD domain of Np95 (mUHRF1) is involved in large-scale reorganization of pericentromeric heterochromatin. Mol. Biol. Cell.

[27] Lorenz D.R., Mikheyeva I.V., Johansen P., Meyer L., Berg A., Grewal S.I., Cam H.P. (2012). CENP-B cooperates with Set1 in bidirectional transcriptional silencing and genome organization of retrotransposons. Mol. Cell. Biol..

[28] Zhu J., Adli M., Zou J.Y., Verstappen G., Coyne M., Zhang X., Durham T., Miri M., Deshpande V., De Jager P.L. (2013). Genome-wide chromatin state transitions associated with developmental and environmental cues. Cell.

[29] Scherthan H., Bähler J., Kohli J. (1994). Dynamics of chromosome organization and pairing during meiotic prophase in fission yeast. J. Cell Biol..

[30] Tanizawa H., Iwasaki O., Tanaka A., Capizzi J.R., Wickramasinghe P., Lee M., Fu Z., Noma K. (2010). Mapping of long-range associations throughout the fission yeast genome reveals global genome organization linked to transcriptional regulation. Nucleic Acids Res..

[31] Osborne C.S., Chakalova L., Brown K.E., Carter D., Horton A., Debrand E., Goyenechea B., Mitchell J.A., Lopes S., Reik W. (2004). Active genes dynamically colocalize to shared sites of ongoing transcription. Nat. Genet..

[32] Lieberman-Aiden E., van Berkum N.L., Williams L., Imakaev M., Ragoczy T., Telling A., Amit I., Lajoie B.R., Sabo P.J., Dorschner M.O. (2009). Comprehensive mapping of long-range interactions reveals folding principles of the human genome. Science.

[33] Dixon J.R., Selvaraj S., Yue F., Kim A., Li Y., Shen Y., Hu M., Liu J.S., Ren B. (2012). Topological domains in mammalian genomes identified by analysis of chromatin interactions. Nature.

[34] Yaffe E., Farkash-Amar S., Polten A., Yakhini Z., Tanay A., Simon I. (2010). Comparative analysis of DNA replication timing reveals conserved large-scale chromosomal architecture. PLoS Genet..

[35] Ryba T., Hiratani I., Lu J., Itoh M., Kulik M., Zhang J., Schulz T.C., Robins A.J., Dalton S., Gilbert D.M. (2010). Evolutionarily conserved replication timing profiles predict long-range chromatin interactions and distinguish closely related cell types. Genome Res..

[36] Dimitrova D.S., Berezney R. (2002). The spatio-temporal organization of DNA replication sites is identical in primary, immortalized and transformed mammalian cells. J. Cell Sci..

[37] Farkash-Amar S., David Y., Polten A., Hezroni H., Eldar Y.C., Meshorer E., Yakhini Z., Simon I. (2012). Systematic determination of replication activity type highlights interconnections between replication, chromatin structure and nuclear localization. PLoS ONE.

[38] Meister P., Taddei A., Ponti A., Baldacci G., Gasser S.M. (2007). Replication foci dynamics: replication patterns are modulated by S-phase checkpoint kinases in fission yeast. EMBO J..

[39] Kind J., Pagie L., Ortabozkoyun H., Boyle S., de Vries S.S., Janssen H., Amendola M., Nolen L.D., Bickmore W.A., van Steensel B. (2013). Single-cell dynamics of genome–nuclear lamina interactions. Cell.

[40] van Koningsbruggen S., Gierlinski M., Schofield P., Martin D., Barton G.J., Ariyurek Y., den Dunnen J.T., Lamond A.I. (2010). High-resolution whole-genome sequencing reveals that specific chromatin domains from most human chromosomes associate with nucleoli. Mol. Biol. Cell.

[41] Olsson I., Bjerling P. (2011). Advancing our understanding of functional genome organisation through studies in the fission yeast. Curr. Genet..

[42] Hou H., Zhou Z., Wang Y., Wang J., Kallgren S.P., Kurchuk T., Miller E.A., Chang F., Jia S. (2012). Csi1 links centromeres to the nuclear envelope for centromere clustering. J. Cell Biol..

[43] Starr D.A., Fridolfsson H.N. (2010). Interactions between nuclei and the cytoskeleton are mediated by SUN–KASH nuclear-envelope bridges. Annu. Rev. Cell Dev. Biol..

[44] Chikashige Y., Yamane M., Okamasa K., Tsutsumi C., Kojidani T., Sato M., Haraguchi T., Hiraoka Y. (2009). Membrane proteins Bqt3 and -4 anchor telomeres to the nuclear envelope to ensure chromosomal bouquet formation. J. Cell Biol..

[45] Fujita I., Nishihara Y., Tanaka M., Tsujii H., Chikashige Y., Watanabe Y., Saito M., Ishikawa F., Hiraoka Y., Kanoh J. (2012). Telomere–nuclear envelope dissociation promoted by Rap1 phosphorylation ensures faithful chromosome segregation. Curr. Biol..

[46] Woolcock K.J., Stunnenberg R., Gaidatzis D., Hotz H.R., Emmerth S., Barraud P., Buhler M. (2012). RNAi keeps Atf1-bound stress response genes in check at nuclear pores. Genes Dev..

[47] Kristell C., Orzechowski Westholm J., Olsson I., Ronne H., Komorowski J., Bjerling P. (2010). Nitrogen depletion in the fission yeast *Schizosaccharomyces pombe* causes nucleosome loss in both promoters and coding regions of activated genes. Genome Res..

